# Long-Term Manure Application Alters *phoC*- and *phoD*-Harboring Bacterial Communities and Soil Phosphatase Activities in Acidic Soil

**DOI:** 10.3390/microorganisms14092107

**Published:** 2026-09-20

**Authors:** Manman Zheng, Long Guo, Zejiang Cai, Chao Wang, Renfang Shen

**Affiliations:** 1 College of Life Science and Agronomy, Zhoukou Normal University, Zhoukou 466001, China; 20191068@zknu.edu.cn; 2 State Key Laboratory of Soil and Sustainable Agriculture, Institute of Soil Science, Chinese Academy of Sciences, Nanjing 210008, China; rfshen@issas.ac.cn; 3 University of Chinese Academy of Sciences, Beijing 100049, China; 4 School of Life and Environmental Science, Wenzhou University, Wenzhou 325035, China; gl19852832129@163.com; 5 Qiyang Red Soil Experimental Station, Chinese Academy of Agricultural Sciences, Qiyang 426182, China; caizejiang@caas.cn

**Keywords:** acidic soil, long-term manure fertilization, organic phosphorus mineralization, *phoC*, *phoD*, phosphatase activity

## Abstract

Long-term manure fertilization changes soil phosphorus (P) availability and the microbial groups involved in organic P mineralization, but *phoC*- and *phoD*-harboring communities may respond differently. In this study, a ten-year continuous field experiment was performed with fertilization regimes: mineral NPK alone and NPK combined with 15, 30, or 45 Mg ha^−1^ year^−1^ fresh pig manure (NPKM1, NPKM2, and NPKM3). Overall, we found that soil pH rose from 4.07 under NPK to 5.24 under NPKM3, and available P increased from 57.4 to 398.6 mg kg^−1^. In addition, acid phosphatase (ACP) activity was higher under NPKM2 and NPKM3, whereas ALP increased significantly only under NPKM3. *phoC* gene abundance was highest under NPKM3, while *phoD* abundance increased progressively across the manure-containing treatments. Diversity and community composition responded differently for the two functional genes. *phoC*-harboring *Cupriavidus* dominated the *phoC* community and was positively associated with ACP activity, whereas *Pleomorphomonas* was positively associated with ALP. Exploratory association networks comprised 85 nodes and 246 edges for *phoC* and 201 nodes and 1093 edges for *phoD*; genus-resolved central OTUs were treated as putative network hubs rather than functional keystones. ACP correlated positively with *phoC* gene abundance (*p* = 0.003) and OTU richness (*p* < 0.001), while ALP correlated strongly with *phoD* gene abundance (*p* < 0.001). Overall, *phoC*- and *phoD*-harboring communities showed distinct responses to long-term fertilization regimes receiving manure, and those responses were associated with phosphatase activity without establishing direct causal pathways.

## 1. Introduction

Acidic soil (pH < 5.5) accounts for more than 50% of the potential arable land globally and is widespread in subtropical southern China [1,2]. The acceleration of soil acidification in many agroecosystems due to the overload of chemical fertilizer and the subsequent depletion of soil fertility within these areas have degraded agricultural production and ecological stability [3,4]. Liming is effective for correcting soil acidity [5,6], whereas manure can also supply organic matter and nutrients and thereby modify several components of soil fertility at the same time [7,8]. Long-term manure application can increase plant-available phosphorus (P) and stimulate P-related enzyme activities, but the biological responses underlying these changes depend on both soil conditions and the fertilization regime [9,10].

Organic P (P_o_) in manure-amended soils must be mineralized before it becomes directly available as inorganic phosphate (P_i_) by soil phosphatases, which are produced principally by soil-phosphorus-mineralizing-related microorganisms [11,12]. Bacterial *phoC* and *phoD* genes, which encode class A nonspecific acid phosphatase (ACP) and [13,14] alkaline phosphatase-producing bacteria (ALP) [15,16], are widely used as credible biomarkers to evaluate phosphorus-mineralizing-related microorganisms. Together, these genes allow acid- and alkaline-phosphatase-associated bacterial groups to be examined separately under contrasting soil pH conditions.

Fertilization can change the abundance, diversity and composition of *phoC*- and *phoD*-harboring communities, and the two gene groups do not necessarily respond in parallel [17,18,19,20,21]. Previous work in acidic soil reported that manure can alter both communities, and long-term organic inputs can also modify their co-occurrence patterns [10,21]. Long-term fertilization studies have also reported management-related shifts in phosphatase-associated microbial groups [9,22,23,24,25]. Field evidence from acidic soils remains limited in three respects: whether *phoC* and *phoD* communities respond differently as manure input increases, which community attributes are most closely associated with ACP and ALP activities, and whether taxa that are central in inferred association networks also track phosphatase activity. Most field studies have focused on *phoD*, leaving *phoC* communities less well characterized [22,23]. A ten-year field experiment in red soil from southern China was performed, and we tested whether *phoC*- and *phoD*-harboring bacterial communities responded differently among mineral NPK and three regimes receiving increasing amounts of pig manure. We quantified phosphatase activities, functional-gene abundance and alpha diversity; compared community composition and exploratory association-network structure; and examined which soil variables and microbial attributes were associated with ACP and ALP activities. The analysis was designed to describe treatment-associated patterns rather than infer direct causal pathways.

## 2. Materials and Methods

### 2.1. Experimental Site and Sampling Collection

The long-term fertilization experiment was established in 2009 at the Red Soil Experimental Station in Qiyang, Hunan Province, China (26°45′ N, 111°52′ E). The site has a humid subtropical monsoon climate, with approximately 1300 mm of annual precipitation, 1470 mm of annual evaporation, a mean daily temperature of 18.0 °C, about 300 frost-free days, and 1610 h of sunshine per year [8]. The soil developed from Quaternary red clay and was classified as Ferralic Cambisol [26]. Maize was grown as a single annual crop. Four fertilization regimes were arranged in a randomized design with three field plots per treatment (13 m × 13 m; 169 m^2^ per plot): (1) NPK, NPK chemical fertilizer only; (2) NPKM1, NPK combined with 15 Mg ha^−1^ year^−1^ fresh pig manure; (3) NPKM2, NPK combined with 30 Mg ha^−1^ year^−1^ fresh pig manure; and (4) NPKM3, NPK combined with 45 Mg ha^−1^ year^−1^ fresh pig manure [8]. Urea, calcium superphosphate, and potassium chloride supplied mineral N, P, and K, respectively. The fresh pig manure contained approximately 70% water. Its mean C concentration was 413.2 g kg^−1^ on a dry-weight basis, whereas N, P, and K concentrations were 3.0, 2.4, and 1.5 g kg^−1^ on a fresh-weight basis, respectively [8]. Total N input was maintained at 225 kg ha^−1^ year^−1^ in each treatment. Accordingly, mineral N was applied at 180, 135, and 90 kg ha^−1^ year^−1^ in NPKM1, NPKM2, and NPKM3, respectively. Mineral P and K inputs were 32.7 and 62.2 kg ha^−1^ year^−1^, respectively. Manure-derived N/P/K inputs were 45/36/22.5, 90/72/45, and 135/108/67.5 kg ha^−1^ year^−1^ in NPKM1, NPKM2, and NPKM3, respectively; therefore, total P and K inputs increased with manure addition [8]. Thirty percent of the mineral fertilizer was applied before sowing, and 70% was applied as topdressing; the full manure dose was incorporated before sowing by rotary tillage.

Soil samples were collected after maize harvest in 2018, following ten annual fertilization and cropping cycles. The field plot was the independent experimental unit (*n* = 3 per treatment). Five cores were collected from the 0–15 cm soil layer in each plot with a 2 cm diameter auger and combined to form one composite sample. The five-core composite was used to integrate within-plot spatial variability, while the same auger diameter and sampling depth were applied consistently across all treatments. Each composite sample was thoroughly homogenized, passed through a 2 mm sieve, and divided into three portions. One portion was frozen immediately at −80 °C for DNA-based analyses, a second was air-dried and finely ground for physicochemical measurements, and a third was kept at 4 °C for no longer than one week before phosphatase activity and $NO_{3}^{-}$-N and $NH_{4}^{+}$-N determinations.

### 2.2. Soil Physicochemical Analyses

Soil pH was determined potentiometrically in a 1:2.5 soil-to-water suspension (*w*/*v*) using a Mettler Toledo FE20 pH meter (Mettler Toledo, Shanghai, China), and soil moisture was measured gravimetrically. Soil total carbon (TC) and total N (TN) were measured using a Vario MAX CNS elemental analyzer (Elementar, Hanau, Germany). Soil total P (TP) and total K (TK) were determined via digestion with HF and HClO_4_, and then TP and TK were determined using the ammonium molybdate ascorbic method and flame photometry (FP640, Shanghai, China) [24], respectively. Soil available P (AP) was extracted with an NH_4_F–HCl solution and quantified calorimetrically using a molybdate–ascorbic acid reaction [21,27]. Soil available K (AK) was extracted using an ammonium acetate solution and determined with flame photometry (FP640, Shanghai, China). Soil $NO_{3}^{-}$-N and $NH_{4}^{+}$-N were extracted using a KCl solution and then determined on a continuous flow analyzer (San^++^, Advanced, Skalar, Holland).

### 2.3. ACP and ALP Activity Analysis

Acid phosphatase (ACP) and alkaline phosphatase (ALP) activities were assayed from the amount of *p*-nitrophenol (*p*NP) released from *p*-nitrophenyl phosphate (*p*NPP), following Tabatabai [28]. Soil was first mixed gently with toluene for 15 min and subsequently incubated for 1 h at 37 °C with pNPP in modified universal buffer adjusted to pH 6.0 for ACP or pH 11.0 for ALP. The reaction was terminated with 0.5 M NaOH, and the filtrate absorbance was recorded at 410 nm with a Spark spectrophotometer (Tecan, Grödig, Austria). Enzyme activity was expressed as μg pNP h^−1^ g^−1^ dry soil.

### 2.4. DNA Extraction and Quantification of Gene Abundance

Soil DNA was extracted from 0.5 g of fresh soil with a Fast^®^DNA SPIN Kit for soil (MP Biomedicals, Santa Ana, CA, USA). Three extraction replicates were prepared from each composite filed sample and pooled before downstream analysis; these extractions were not treated as independent field replicates. The pooled DNA was purified with a PowerClean DNA Clean-Up Kit (Mobio, San Mateo, CA, USA) and quantified with a NanoDrop ND-1000 spectrophotometer (NanoDrop Technologies, Wilmington, NC, USA).

The primer pair phoc-A-F1 (5′-CGGCTCCTATCCGTCCGG-3′)/phoc-A-R1 (5′-CAACATCGCTTTGCCAGTG-3′) [13,14], designed to target class A bacterial nonspecific acid phosphatase genes, was used for *phoC*-harboring bacteria quantification. ALPS-F730 (5′-CAGTGGGACGACCACGAGGT-3′)/ALPS-R1101 (5′-GAGGCCGATCGGCATGTCG-3′) [29,30] was used for *phoD*-harboring bacteria. Quantitative PCR was conducted in a LightCycler 480 real-time PCR system (Roche Diagnostics, Mannheim, Germany) using SYBR^®^Premix Ex Taq™ (TaKaRa Bio, Dalian, China). Reaction mixtures, cycling conditions, and gene-fragment standard curves followed Zheng et al. [31]. Each field-sample DNA extract was analyzed in three qPCR technical replicates. Amplification efficiencies were 98.2% for *phoC* (R^2^ = 0.99) and 105.5% for *phoD* (R^2^ = 0.98).

### 2.5. High-Throughput Sequencing and Data Processing

The phoc-A-F1/phoc-A-R1 [13] and ALPS-F730/ALPS-R1101 [29] primer pairs were used to amplify *phoC* and *phoD*, respectively. A sample-specific 7 bp barcode was attached to each forward primer. Three PCR amplifications per sample were combined, purified, normalized to equal molar concentrations, and pooled. The *phoC* library was sequenced as paired-end 150 bp reads on an Illumina HiSeq platform, whereas the *phoD* library was sequenced as paired-end 250 bp reads on an Illumina MiSeq platform (Shanghai Personal Biotechnology Co., Ltd., Shanghai, China). The two gene datasets were processed independently throughout the bioinformatic workflow. The *phoC* and *phoD* sequencing data were deposited in the NCBI Sequence Read Archive (SRA) database under BioProject accession numbers PRJNA1501897 and PRJNA1501898, respectively.

Paired reads were merged with FLASH (version 1.2.7) [32] and processed in QIIME v1.8.0 [33]. Reads shorter than 130 bp for *phoC* or 150 bp for *phoD*, reads containing ambiguous nucleotides, and reads that did not match the primer sequence were removed. Chimeric sequences were identified and removed with USEARCH v5.2.236 (http://www.drive5.com/usearch/; accessed on 8 July 2019). The remaining high-quality sequences were clustered into operational taxonomic units (OTUs) at 97% sequence similarity using UCLUST [34]. Representative OTU sequences were assigned taxonomically by BLAST searches against the NCBI nucleotide database using an E-value cutoff of 0.001. Samples were rarefied to 18,877 reads for *phoC* and 8399 reads for *phoD* before downstream analyses. After quality filtering, 1,126,367 *phoC* reads (32,120–128,998 per sample) and 370,144 *phoD* reads (24,926–36,150 per sample) were retained. Per-sample OTU richness ranged from 86 to 302 for *phoC* and from 641 to 1361 for *phoD* (Table S1).

### 2.6. Network Analyses

Association networks for *phoC*- and *phoD*-harboring bacterial OTUs were constructed separately with the CoNet plugin in Cytoscape v3.2.1. OTUs detected in fewer than 6 of the 12 samples were removed during preprocessing (row-minocc = 6), and column-wise normalization was applied. Edge stability was evaluated using 100 permutation iterations followed by 100 bootstrap iterations. Unstable edges were filtered. The false discovery rate was controlled using the Benjamini–Hochberg procedure, and associations with adjusted *p* values < 0.05 were retained [35]. The final exported networks contained Spearman-based associations and were treated as undirected networks. Network topology was recalculated from the final edge lists using NetworkX v3.6.1, and the revised networks were plotted with Matplotlib v3.10.8. Reported global descriptors included the node and edge numbers, proportions of positive and negative associations, network density, mean degree, and mean clustering coefficient. Putative network hub OTUs were operationally defined as nodes with both degree and betweenness centrality at or above the 90th percentile and detection in at least 6 of the 12 samples. For taxonomic interpretation in network, only hub candidates resolved to the genus level were listed and highlighted. Hub designation reflects topological centrality within the inferred association network and does not imply a functionally validated keystone role.

### 2.7. Statistical Analyses

Treatment effects were evaluated at the field-plot level, with three independent plots per fertilization regime. Data were analyzed by one-way analysis of variance (ANOVA) in SPSS v20.0 (IBM Corp., Armonk, NY, USA). Normality of ANOVA residuals and homogeneity of variance were rechecked using the Shapiro–Wilk test and Levene’s test, respectively; neither test indicated a significant departure from the assumptions for the variables analyzed (*p* > 0.05; Table S2). No data transformations were applied before ANOVA. When the omnibus ANOVA was significant, treatment means were separated using Duncan’s multiple-range test, with *p* < 0.05 considered significant. Spearman’s correlation was used to examine relationships among soil properties, gene copy numbers, alpha-diversity indices, relative abundances of bacterial genera, and phosphatase activities.

OTU richness and the Shannon index were calculated in QIIME. Principal coordinate analysis (PCoA) was used to visualize differences in community composition among samples, and analysis of similarities (ANOSIM) tested separation between NPK and the manure-containing fertilization regimes. PCoA and ANOSIM were based on Bray–Curtis dissimilarities calculated from the rarefied OTU data and implemented with the vegan package in R v2.15.0.

Each manure-containing treatment was compared separately with NPK to describe endpoint OTU detection patterns. An OTU was considered detected when it occurred in at least one of the three independent field plots within a treatment and undetected only when it was absent from all three plots. OTUs were classified as shared (detected in both treatments), manure-associated gains (detected only in the manure-containing treatment), or NPK-associated losses (detected only in NPK). OTUs absent from both treatments were excluded from the denominator, and each category was expressed as a proportion of the sum of the three detected categories. Because the communities were sampled once, these categories were treated strictly as detection patterns rather than evidence of immigration, extinction, or resuscitation.

## 3. Results and Discussion

### 3.1. Soil Physicochemical Properties, Phosphatase Activities and Gene Copy Number

Fertilization significantly affected several analyzed soil properties (Table 1). Compared with the NPK treatment, the increased soil pH and TP, AP, and $NO_{3}^{-}$-N content, and decreased $NH_{4}^{+}$-N content, C/P and N/P were observed under all manure addition treatments (*p* < 0.05), except the $NO_{3}^{-}$-N content under NPKM3 did not reach statistical significance.

**Figure 1:**
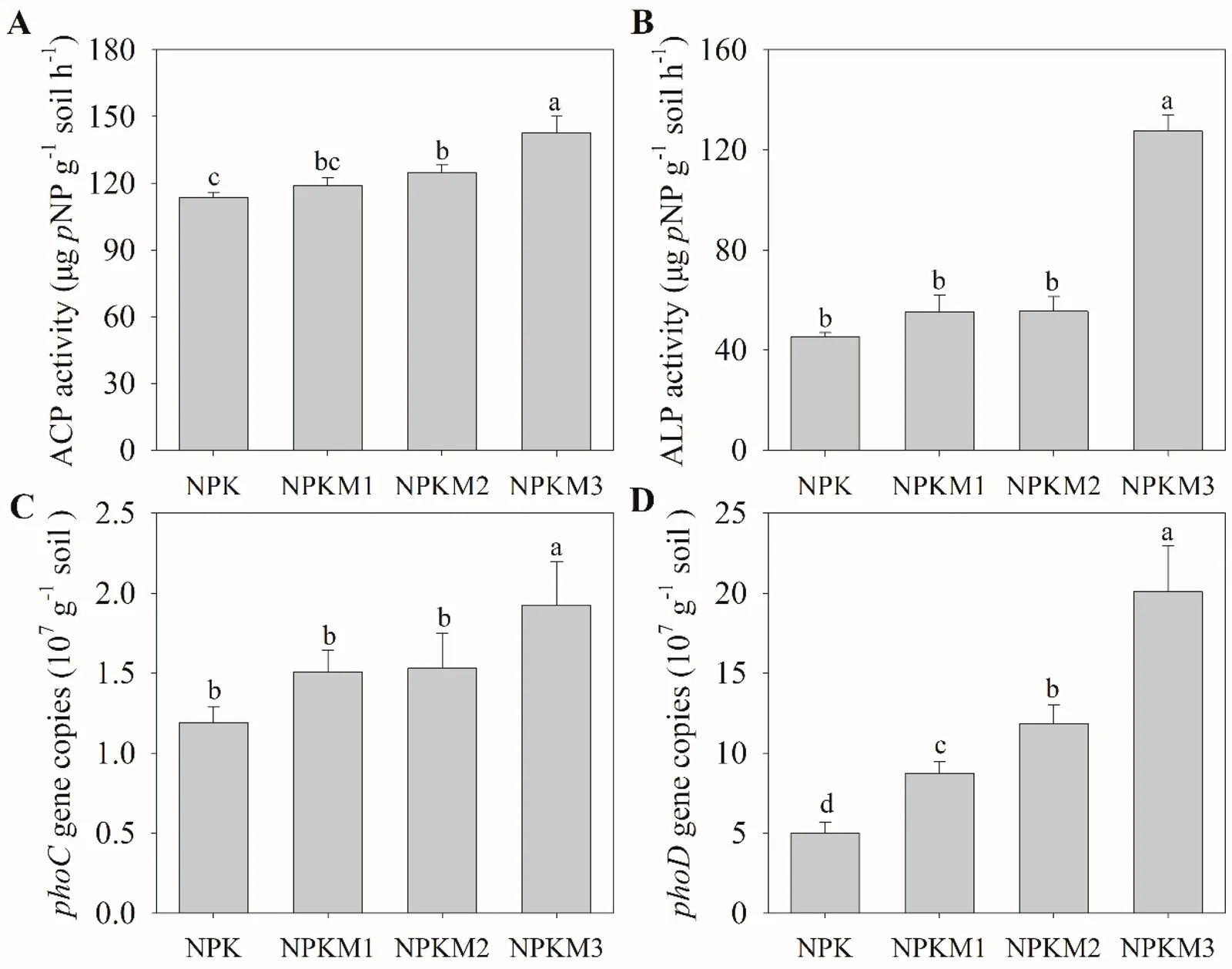
ACP activity (**A**), ALP activity (**B**) and *phoC* (**C**) and *phoD* (**D**) gene copy number under the four fertilization regimes after ten annual fertilization and cropping cycles. Values are presented as the mean ± standard deviation (*n* = 3 independent field plots). Different lowercase letters above the columns indicate significant differences among fertilizer treatments at *p* < 0.05.

Importantly, the increased soil pH after OM application, especially in the NPKM3 treatment (increased by 28.83%), was accompanied with increased ACP by 25.61% and improved ALP by 182.23% (Figure 1A,B), as ACP activity generally prevails in acidic soils, whereas ALP activity prevails in alkaline soil environment [12]. Therefore, a substantial increase in soil pH significantly promotes ALP activity.

For phosphatase, NPKM2 and NPKM3 markedly (*p* < 0.05) promoted ACP activity, while only NPKM3 sharply increased ALP activity (Figure 1A,B), revealing that ACP was more susceptible to organic manure addition than ALP in acidic soil, as reported previously [21]. Additionally, we found that *phoC* and *phoD* gene copy numbers were both increased with the increasing dose of the organic manure application rate (Figure 1C,D), which may be caused by the improved soil pH and available nutrition (e.g., TC and AP) after organic matter application. Similar to our results, Liu et al. [36] also reported that organic fertilization elevates soil labile and total organic carbon pools, which enriches the overall soil microbial community, including *phoD*-carrying microorganisms. The strong positive correlation between soil pH, available nutrition and gene copies of *phoC*- and *phoD*-harboring bacteria (Figure S1) also explained our hypothesis. However, opposite results were observed in Luo et al. [10], who found that organic-only fertilizer and a combination of mineral and organic fertilizer amendments both showed no effect on *phoC* gene abundance. The contradictory results are most likely attributable to the strongly acidic soil used in this experiment, as organic fertilizers can readily stimulate microbial growth by altering soil pH or nutrient availability.

Similar to Guo et al. [37] and Li et al. [38], our results found that ALP activity and *phoD* abundance did not change in parallel across all treatments (Figure 1B,D), indicating that ALP activity is not likely to be tightly controlled by the *phoD* gene abundance. On the one hand, ALP activity may not be strictly dependent on viable microbial biomass, as extracellular soil enzymes can remain catalytically active after microbial cells are inactivated [39,40]; thus, the phosphatase activity we measured cannot be identified as the direct source of bulk-soil enzyme activity. On the other hand, the *phoD* gene abundances do not accordingly indicate the real-time transcription and translation level [38].

In contrast, we found that the *phoD* gene abundance was positively correlated with ALP activity (Figure S1), indicating that *phoD* gene abundance acts as a potential critical role in elevating ALP activity. This phenomenon may be attributed to the NPKM3 treatment, which drastically increased both *phoD* gene abundance and ALP activity, thereby raising the contribution proportion of gene abundance to enzyme activity. Further comprehensive analysis was conducted to clarify the dominant factors governing the enhancement of ALP activity.

### 3.2. phoC- and phoD-Harboring Bacterial Community

**Figure 2:**
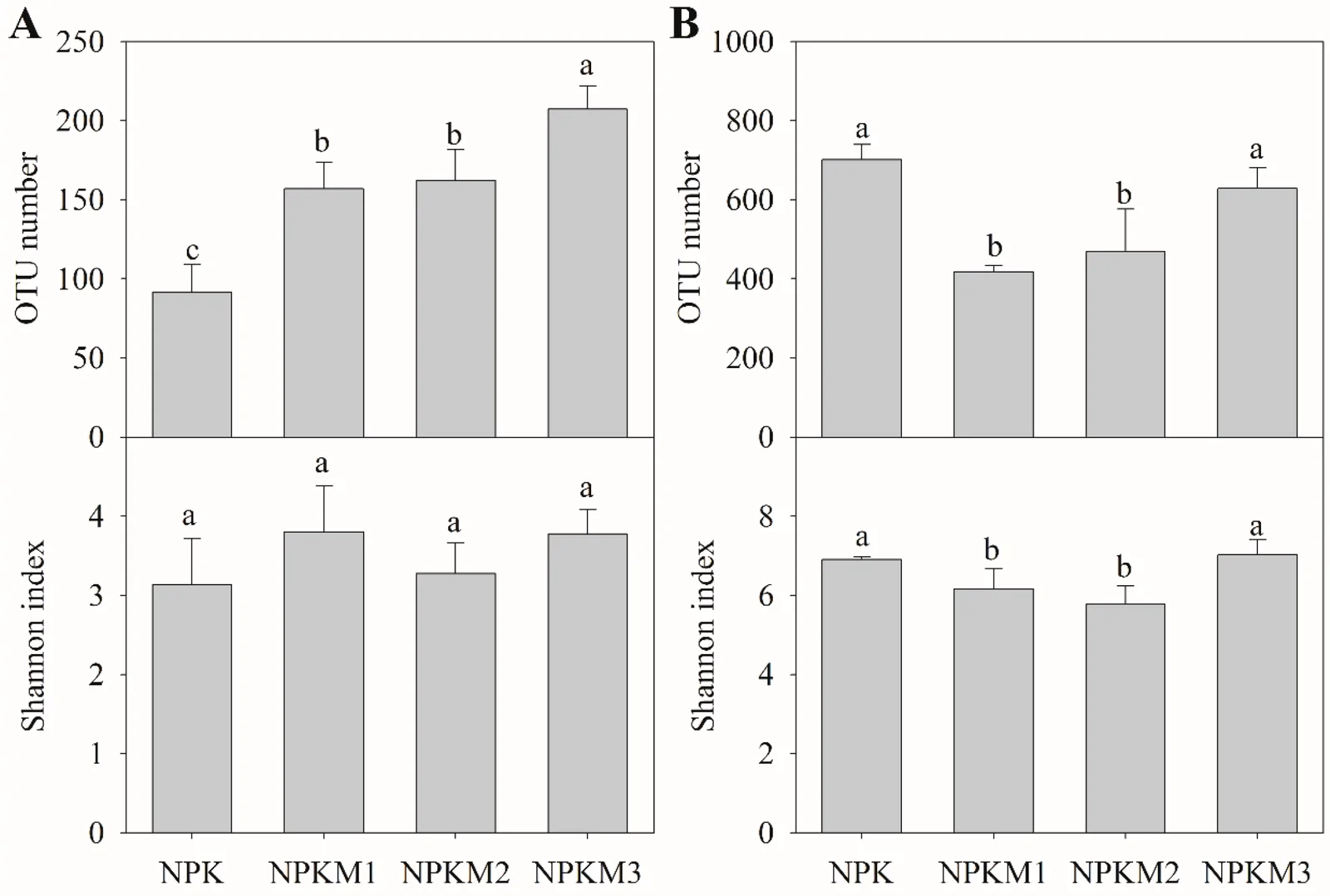
The OTU number and Shannon index of *phoC*- (**A**) and *phoD*- harboring (**B**) bacteria in soils with different long-term (10 years) manure addition treatments. Values are presented as the mean ± SD of three plot replicates. Different lowercase letters above the columns indicate significant differences among fertilizer treatments at *p* < 0.05.

**Figure 3:**
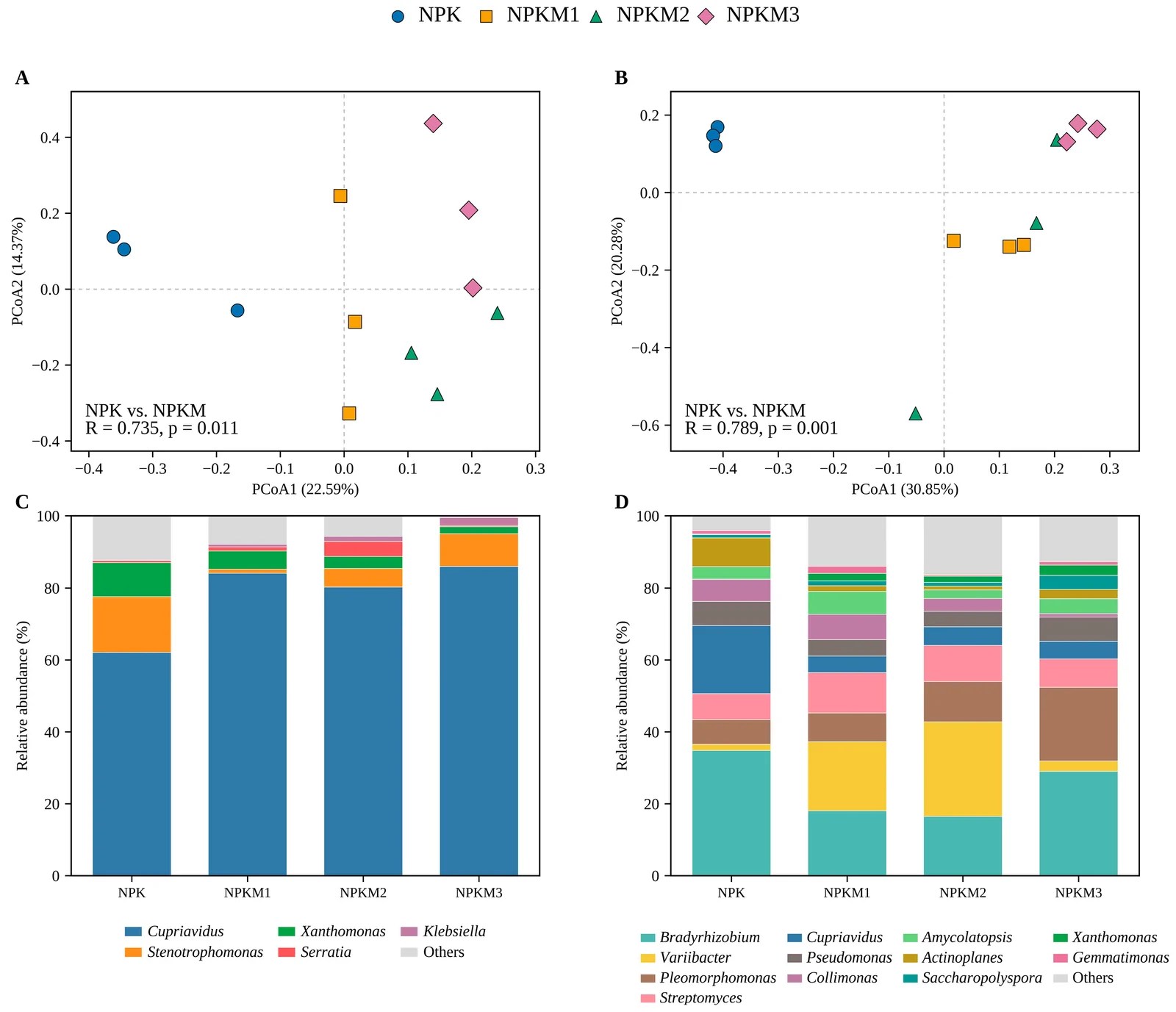
Principal coordinate analysis (PCoA) of community composition and relative abundances (%) of the most abundant genera (>1%) of *phoC*- (**A**,**C**) and *phoD*- harboring (**B**,**D**) bacteria across all of the soil samples with different long-term (10 years) manure addition treatments. The differences in *phoC* and *phoD* harboring bacterial community composition between manure addition treatment and NPK treatment by ANOSIM.

Similar to gene copy number, the OTU number of *phoC*-harboring bacteria was significantly increased (*p* < 0.05, Figure 2A) under manure-containing fertilizer treatments compared to NPK and was strongly correlated with soil pH and available nutrition (e.g., TC and AP) (Figure S1), speculating that the increasing gene copy numbers may have resulted from the increasing OTU numbers. Bacteria encoding the *phoC* gene are generally classified as oligotrophic K-strategists [41]. The rise in OTU number is likely driven by the resuscitation of dormant microbial populations, as supported by the elevated relative abundances of taxa that were originally extremely rare, such as *Serratia* and *Klebsiella* (Figure 3C).

The *phoC*-harboring *Cupriavidus* was dominant in all treatments and considerably increased by 29.22% to 38.30% under organic manure addition (Figure 3C), underling its essential role in modulating ACP activity. *Cupriavidus* was reported as the dominant *phoC*-harboring bacterial genus in acidic soils due to its strong resistance to metal toxicity such as aluminum (Al) by expressing relevant resistance genes, which may help adapt to the acidic soil with high Al toxicity [31,42]. Our result also found that the abundance of *Cupriavidus* was markedly positive with ACP activity (*p* < 0.05, Table S4), confirming its potential contributing role in producing ACP.

Opposite to the results of *phoD* gene copy numbers and ALP activities, the OTU number and Shannon index of *phoD*-harboring bacteria were significantly reduced under NPKM1 and NPKM2. (*p* < 0.05, Figure 2B, and were not increased under NPKM3 compared with NPK (Figure 2B). This contrasted with the progressive increase in *phoD* gene abundance and the marked rise in ALP activity under NPKM3. These results were similar to Wang et al. [43], who reported that long-term organic fertilization markedly improved ALP activity but not community richness or the Shannon index. A previous study has shown that ALP activity was determined by specific microorganisms rather than their diversity [44]. In the present study, *phoD*-harboring *Variibacter* and *Streptomyces* were sharply increased by 1008.44% and 55.52% under NPKM1 and 1414.96% and 39.47% under NPKM2, respectively, and *phoD*-harboring *Pleomorphomonas* was remarkedly promoted in NPKM3 treatment by 199.12% (Figure 3D), suggesting its potential role in secreting ALP at different organic fertilizer application rate. The genera *Streptomyces*, *Variibacter* and *Pleomorphomonas* were detected as reliable P_o_ mineralizers, with crucial roles in increasing phosphatase activity [20,43]. The relative analysis showed that the relative abundance of *Pleomorphomonas* was positively linked with ALP activity (*p* < 0.05, Table S4), highlighting its potential function for releasing ALP. Therefore, these results verified that ALP thus tracked *phoD* gene abundance and the abundance of particular taxa more closely than alpha diversity. In addition, Figure S1 also shows weak relationships between most soil properties and *phoD*-harboring OTU richness or Shannon diversity (Figure S1). The r-strategic *phoD*-harboring bacteria are highly susceptible to short-term nutrient pulses, interspecific competition, and antagonistic inhibition. The drastic community simplification and dominant genus monopolization under long-term organic manure input may substantially increase the stochasticity of *phoD* community assembly, ultimately weakening their linear correlations with soil environmental variables [45].

Interestingly, the *phoD*-harboring bacteria *Cupriavidus* decreased drastically by nearly 73% following all organic manure amendment treatments (Figure 3D), which might be derived from the different primer pairs and sequencing platforms between *phoC* and *phoD.* Nevertheless, these findings also suggest, to a certain degree, that these microorganisms shift their metabolic focus toward ACP production upon manure input, with a concurrent suppression of ALP secretion [41]. The performance of P_o_-mineralizing functions by distinct microbial genera in differentiated habitats represents a vital survival and adaptation strategy for microorganisms, which enables microbial groups with diverse physiological traits to maximize their P_o_-mineralizing efficiency in specific niches.

However, it should be acknowledged that soil enzymes originate from multiple sources, including microorganisms, plant roots, and enzymes associated with mineral particles [12]. Hence, our inferences regarding the contributions of specific microorganisms to enzyme activity are only suggestive, not conclusive. Consequently, the specific microbial genera identified as phosphatase producers in our study require additional experimental validation to confirm their functional significance.

Principal coordinate analysis (PCoA) showed that NPK samples were separated from the manure-containing treatments for both functional-gene communities (Figure 3A,B). ANOSIM confirmed significant separation for *phoC* (R = 0.735, *p* = 0.011) and *phoD* (R = 0.789, *p* = 0.001). Comparable shifts in *phoC*- and *phoD*-harboring communities under long-term fertilization have also been reported by previous studies [21,37].

The community structure of *phoC*-harboring bacteria was uniquely shaped by large amounts of TC and TN introduced by organic fertilizer, whereas the *phoD*-encoding microbial community structure was distinctively governed by organic-fertilizer-induced shifts in soil pH (Figure S1). Numerous studies have reported that ACP activity generally prevails in acidic soils, whereas ALP activity prevails in alkaline soils [12]. The soil pH ranges from 4.07 to 5.24 in the present study (Table 1), a suitable acidic environment for microorganisms that produce ACP to exert their ecological functions. Accordingly, abundant carbon and nitrogen inputs derived from organic fertilizer became key factors reshaping the community structure of these microbes. By contrast, ALP-producing taxa faced a metabolically suboptimal environment, where the pH elevation induced by organic fertilization became the overriding factor governing their community assembly.

### 3.3. Association-Network Structure of phoC- and phoD-Harboring Bacterial Communities

**Figure 4:**
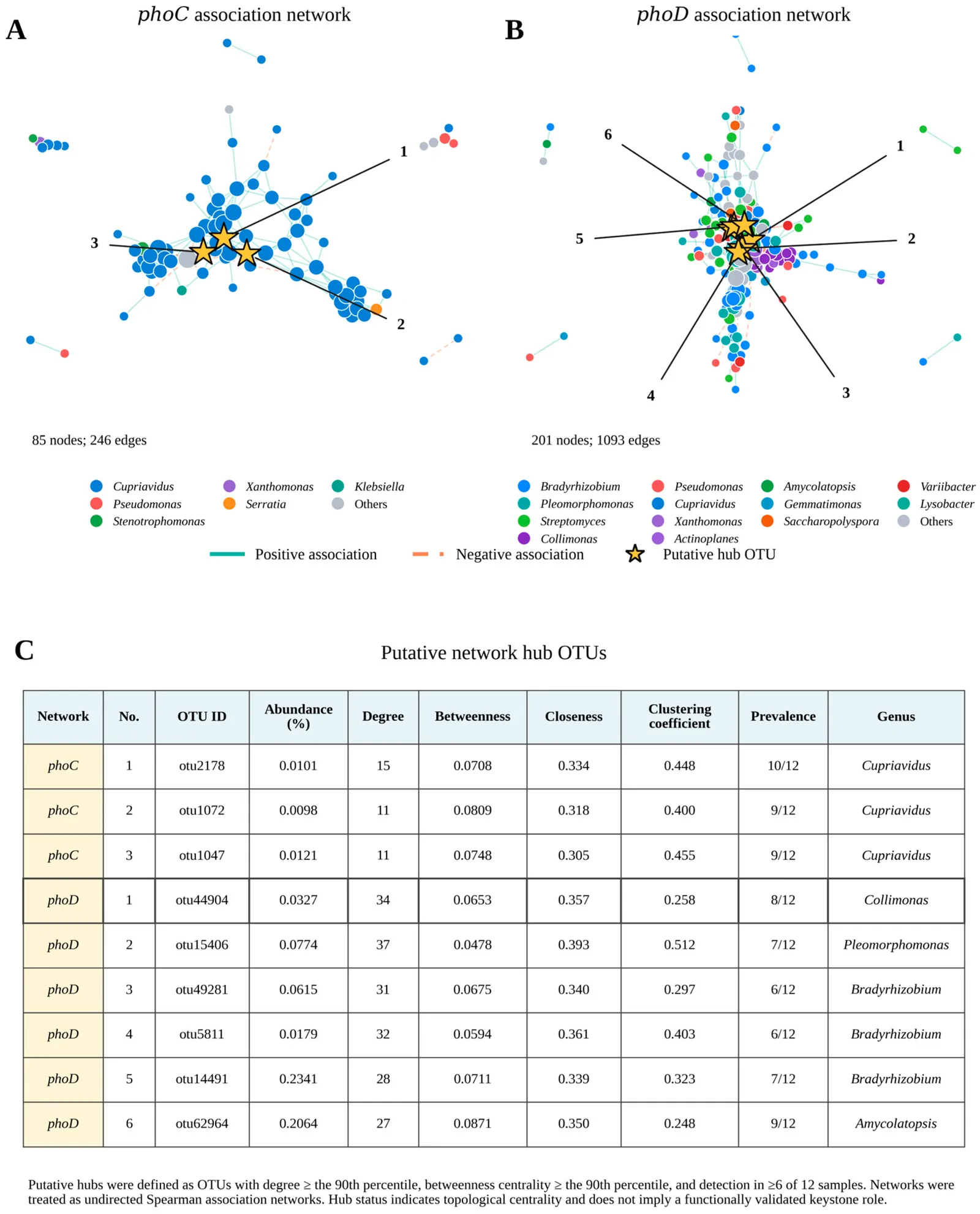
Global association networks and genus-resolved putative hub OTUs of *phoC*- and *phoD*-harboring bacterial communities. (**A**) *phoC* and (**B**) *phoD* undirected Spearman association networks. Solid and dashed edges denote positive and negative associations, respectively; node size reflects degree, and node color denotes genus-level taxonomy. Starred and numbered nodes denote hub candidates with genus-level assignments. (**C**) Topological characteristics of the genus-resolved putative hub OTUs. Putative hubs were defined as OTUs with degree and betweenness centrality at or above the 90th percentile and detection in at least 6 of 12 samples.

Two association networks were established based on the correlations among *phoC*- and *phoD*-harboring bacterial OTUs, respectively (Figure 4A,B and Figure S3). The *phoC* network contained 85 nodes and 246 edges, of which 216 (87.80%) were positive and 30 (12.20%) were negative associations. The *phoD* network contained 201 nodes and 1093 edges, including 600 (54.89%) positive and 493 (45.11%) negative associations. The network density, mean degree, and mean clustering coefficient were 0.069, 5.788, and 0.391 for *phoC* and 0.054, 10.876, and 0.437 for *phoD*, respectively.

Interestingly, *phoC*-harboring bacteria exhibited a higher percentage of positive edges than *phoD* (Table S3). As K-strategists, *phoC*-harboring microbes possess specialized metabolic capabilities and may depend on complementary metabolic exchanges to mineralize recalcitrant P_o_ derived from organic fertilizers. Consistent with this interpretation, slower-growing, stress-tolerant microorganisms can promote interspecific functional coupling through the release and cross-feeding of public goods [46]. In contrast, copiotrophic *phoD* hosts can rapidly exploit labile low-molecular-weight substrates released during organic matter decomposition, potentially reducing their dependence on such exchanges, which may explain the higher proportion of negative associations in the *phoD* network.

Among the putative *phoC* hub candidates, three OTUs had genus-level assignments, all affiliated with *Cupriavidus*: otu2178, otu1072 and otu1047 (Figure 4C, Table S7). They were detected in 9–10 of the 12 samples and ranked highly for both degree and betweenness centrality. The repeated occurrence of *Cupriavidus* among central nodes agrees with its dominance in the *phoC* community and its positive association with ACP activity (Table S4), confirming its essential role in P_o_ mineralization.

For the putative *phoD* hub candidates, six OTUs had genus-level assignments: otu44904 (*Collimonas*), otu15406 (*Pleomorphomonas*), otu49281, otu5811 and otu14491 (*Bradyrhizobium*), and otu62964 (*Amycolatopsis*) (Figure 4C, Table S7). This indicated a speculated potential functional role. Additionally, *Collimonas* strains can solubilize P_i_ and produce gluconic acid from glucose, demonstrating that acidification is one of the key mechanisms conducted by these bacteria for mineral weathering as well [47]. We therefore treat these OTUs as candidates for functional validation rather than confirming a functionally validated keystone role.

Importantly, we clearly state that while our results demonstrated enhanced microbial P mineralization (increased phosphatase) and greater AP in the soil, the subsequent transfer and accumulation of this mineralized P in maize plants remains to be confirmed. The isotopic tracing (e.g., ^33^P labeling) or plant tissue analysis is necessary to track the actual fate of bio-released P in crop systems [48], which will be a critical next step for validating the agronomic value of these microbial shifts.

### 3.4. Treatment-Specific OTU Detection Patterns

Treatment-level detection contrasts revealed different patterns in shaping *phoC*- and *phoD*- harboring bacterial communities. For *phoC*-harboring bacteria, relative to NPK, manure-associated gains accounted for 55.2%, 51.8% and 61.1% of the detected OTU pool in NPKM1, NPKM2 and NPKM3, respectively, whereas NPK-associated losses accounted for 21.2%, 22.3% and 21.1%. Shared OTUs represented 23.6%, 25.9% and 17.8% of the corresponding comparison pools. Thus, *phoC* comparisons consistently contained a larger fraction of OTUs detected only under the manure-containing regime than OTUs detected only under NPK. As oligotrophic microorganisms, *phoC*-harboring bacteria are conventionally expected to thrive under low-nutrient conditions and generally exhibit high physiological tolerance to osmotic and acidic stresses, while the buffered nature of organic amendments avoids acute environmental filtering. Therefore, the observed pattern should be interpreted as a “resuscitation and expansion” rather than a competitive turnover of the *phoC* guild [49].

**Figure 5:**
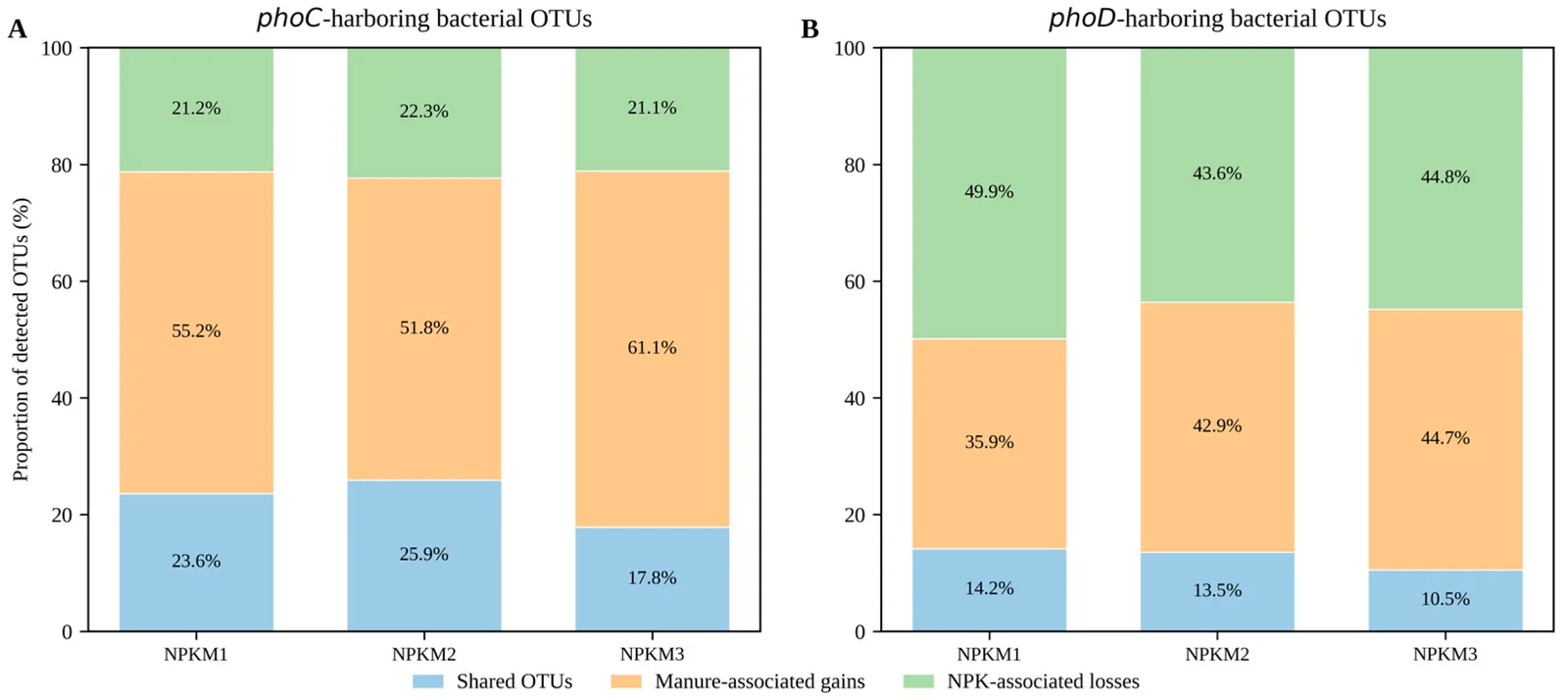
Treatment-specific OTU detection patterns for *phoC*- (**A**) and *phoD*-harboring (**B**) bacterial communities. Each manure-containing treatment was compared separately with NPK. Shared OTUs were detected in both treatments; manure-associated gains were detected only in the manure-containing treatment; and NPK-associated losses were detected only in NPK. OTUs undetected in both treatments were excluded from the denominator. Presence was defined at the treatment level as detection in at least one of the three independent field plots.

The *phoD* detection pattern differed from *phoC* (Figure 5B). Shared OTUs accounted for 14.2%, 13.5% and 10.5% of the NPKM1, NPKM2 and NPKM3 comparison pools, respectively. Manure-associated gains represented 35.9%, 42.9% and 44.7%, while NPK-associated losses represented 49.9%, 43.6% and 44.8%. The high gain and loss fractions showed that many *phoD* OTUs were treatment-specific. These results may be largely attributed to the alleviation of carbon limitation and the shift in substrate complexity. The exclusive application of chemical fertilizers sustains a simplified, oligotrophic community adapted to labile inorganic nutrients. Conversely, the incorporation of organic manure introduces diverse polymeric organic compounds [50], exerting a strong selective pressure that favors copiotrophic taxa with enhanced capabilities for degrading complex biopolymers (e.g., cellulose and phytate) while simultaneously displacing specialist oligotrophs that fail to compete for newly available niches. Thus, the disappearance and the emergence of certain OTUs represent a classic ecological response to resource-mediated environmental filtering rather than a mere stochastic event.

However, several limitations should be considered when interpreting the present results. On the one hand, the experiment was conducted at a single site and sampled once after harvest, with three independent field plots per treatment. Future studies will incorporate multi-site and continuous seasonal sampling to verify the universality and temporal stability of the findings, further strengthening the reliability of the relevant conclusions. On the other hand, the ALPS-F730/ALPS-R1101 primer pair may preferentially recover some Alphaproteobacterial *phoD* sequences and does not capture the complete soil *phoD* pool [51], while the *phoC* primers target class A bacterial nonspecific acid phosphatase genes rather than all acid phosphatase producers. Additionally, the different sequencing platforms of *phoC* (HiSeq, 150 bp PE) and *phoD* (MiSeq, 250 bp PE) may introduce technical biases that complicate direct cross-gene comparisons. Consequently, we acknowledge that certain functionally relevant phylotypes—particularly those harboring divergent *phoD* or *phoC* alleles—may have been underrepresented or overlooked in our analyses. Future studies employing degenerate primers or shotgun metagenomic sequencing could mitigate this bias and capture a broader spectrum of *phoC*- and *phoD*-encoding phylotypes, thereby facilitating the rational harnessing of these microbial functions for improved phosphorus management in acidic soils.

## 4. Conclusions

In conclusion, ten years of manure application increased soil pH and P availability and altered both phoC- and phoD-harboring communities. ACP activity increased with manure and strongly correlated with phoC gene abundance and richness; ALP increased only under NPKM3 and markedly correlated with phoD abundance. In addition, NPKM3 not only triggered a stronger ALP response but also led to more pronounced shifts in OTU composition and network connectivity. Cupriavidus and Pleomorphomonas were key taxa for ACP and ALP, respectively, and occupied central network positions, indicating their potential role in P_o_ mineralization. Notably, the two functional-gene datasets exhibited contrasting patterns of treatment-specific OTU occurrence. However, as our findings derive from a single acidic red soil under specific fertilization practices, their generalizability to other soil types or alternative management strategies remains uncertain; further studies across diverse agro-ecological conditions are required to validate the functional contributions of the candidate taxa identified herein.

## Figures and Tables

**Table 1:** Soil properties and soil C:N:P stoichiometry of different long-term (10 years) manure addition treatments.

Soil Properties	NPK	NPKM1	NPKM2	NPKM3
pH	4.07 ± 0.16 c	4.36 ± 0.07 b	4.58 ± 0.11 b	5.24 ± 0.19 a
TC (g kg^−1^)	9.25 ± 0.30 b	10.97 ± 0.85 a	10.37 ± 0.36 ab	11.10 ± 0.72 a
TN (g kg^−1^)	1.22 ± 0.18 a	1.39 ± 0.17 a	1.38 ± 0.03 a	1.47 ± 0.13 a
$NH_{4}^{+}$-N (mg kg^−1^)	8.26 ± 0.43 a	7.11 ± 0.44 b	5.84 ± 0.59 c	5.60 ± 0.26 c
$NO_{3}^{-}$-N (mg kg^−1^)	20.51 ± 2.47 c	29.89 ± 3.67 a	27.78 ± 3.45 ab	22.14 ± 2.37 bc
TP (g kg^−1^)	0.58 ± 0.06 c	0.98 ± 0.07 b	1.09 ± 0.13 b	1.34 ± 0.16 a
AP (mg kg^−1^)	57.41 ± 4.27 d	213.96 ± 19.79 c	264.72 ± 26.56 b	398.57 ± 28.43 a
TK (g kg^−1^)	11.63 ± 0.63 a	11.76 ± 1.18 a	13.43 ± 1.48 a	12.12 ± 1.34 a
AK (mg kg^−1^)	232.71 ± 15.71 b	259.97 ± 21.13 b	219.18 ± 9.80 b	333.12 ± 48.98 a
C/N	7.69 ± 1.06 a	7.95 ± 0.95 a	7.52 ± 0.25 a	7.58 ± 0.20 a
C/P	15.96 ± 1.79 a	11.29 ± 1.76 b	9.55 ± 0.80 bc	8.32 ± 0.45 c
N/P	2.09 ± 0.24 a	1.43 ± 0.22 b	1.27 ± 0.12 b	1.10 ± 0.03 b

Data are means ± standard deviation (*n* = 3 independent field plots). Different lowercase letters within rows indicate significant differences among fertilization treatments at *p* < 0.05. TC: soil total carbon; TN: total nitrogen; $NH_{4}^{+}$-N: ammonium nitrogen; $NO_{3}^{-}$-N: nitrate nitrogen; TP: total phosphorus; AP: available phosphorus; TK: total potassium; AK: available potassium; C/N ratio: soil total carbon/total nitrogen; C/P ratio: soil total carbon/total phosphorus; N/P ratio: total nitrogen/total phosphorus.

## Data Availability

The raw data supporting the conclusions of this article will be made available by the authors on request.

## Supplementary Materials

The following supporting information can be downloaded at https://www.mdpi.com/article/10.3390/microorganisms14092107/s1, Table S1. Summary of usable sequences after quality filtering and operational taxonomic units (OTUs). Table S2. Overall one-way ANOVA and assumption-check statistics for variables analyzed across fertilization treatments. Table S3. Global topological properties of the *phoC* and *phoD* association networks. Table S4. The correlation between soil properties, phosphatase activities and dominant *phoC*- and *phoD*-harboring bacterial genera. Only data with significant correlation relationships are retained. Table S5. The values of ACP and ALP activity, *phoC* and *phoD* gene copy number, OTU number and Shannon index of *phoC*- and *phoD*- harboring bacteria in soils with different long-term (10 years) manure addition treatments. Table S6. The coordination of PCoA of *phoC*- and *phoD*-harboring bacteria. Table S7. The putative network hub of *phoC*- and *phoD*-harboring bacteria. Figure S1 Correlation analysis between soil properties, phosphatase activities, gene abundance, α-diversity (OTU number and Shannon index), and bacterial community composition (PCoA1) were evaluated using Spearman’s correlation. * *p* < 0.05; ** *p* < 0.01; *** *p* < 0.001. TC: soil total carbon; TN: total nitrogen; $NH_{4}^{+}$-N: ammonium nitrogen; $NO_{3}^{-}$-N: nitrate nitrogen; TP: total phosphorus; AP: available phosphorus; TK: total potassium; AK: available potassium. C/N ratio: soil total carbon/total nitrogen; C/P ratio: soil total carbon/total phosphorus; N/P ratio: total nitrogen/total phosphorus.

## Abbreviations

The following abbreviations are used in this manuscript:

ACP	Acid phosphatase
ALP	Alkaline phosphatase
TC	Soil total carbon
TN	Total nitrogen
$NH_{4}^{+}$-N	Ammonium nitrogen
$NO_{3}^{-}$-N	Nitrate nitrogen
TP	Total phosphorus
AP	Available phosphorus
TK	Total potassium
AK	Available potassium
C/N ratio	Soil total carbon/total nitrogen
C/P ratio	Soil total carbon/total phosphorus
N/P ratio	Total nitrogen/total phosphorus
