# Supplementary material for: Long-Term Manure Application Alters phoC- and phoD-Harboring Bacterial Communities and Soil Phosphatase Activities in Acidic Soil
Source: Microorganisms. 2026 Sep 20;14(9):2107. doi: 10.3390/microorganisms14092107 (PMC13608974; doi:10.3390/microorganisms14092107)
Supplement: Supplementary file 1 [file microorganisms-14-02107-s001.zip › microorganisms-4506407-supplementary.pdf]

microorganisms

Article

Long term Manure application Alters *phoC*- and *phoD*-harboring bacterial communities and Soil Phosphatase Activities in acidic soil

Manman Zheng[1,2,3†], Long Guo[4†], Zejiang Cai[5], Chao Wang[2,3]* and Renfang Shen[2,3]

1 College of Life Science and Agronomy, Zhoukou Normal University, Zhoukou, China; 20191068@zknu.edu.cn
2 State Key Laboratory of Soil and Sustainable Agriculture, Institute of Soil Science, Chinese Academy of Sciences, Nanjing 210008, China; chwang@issas.ac.cn; rfshen@issas.ac.cn
3 University of Chinese Academy of Sciences, Beijing 100049, China
4 School of Life and Environmental Science, Wenzhou University, Wenzhou 325035, Zhejiang, China; gl19852832129@163.com
5 Qiyang red soil experimental station, Chinese Academy of Agricultural Sciences, Qiyang 426182, China; caizejiang@caas.cn

* Correspondence: Chao Wang; chwang@issas.ac.cn; Tel: +86-25-86881578; Fax: +86-25-86881000

Table S1. Summary of usable sequences after quality filtering and operational taxonomic units (OTUs).

Treatment	*phoC*-harboring bacteria		*phoD*-harboring bacteria	
	Sequences numbers	OTU numbers	Sequences numbers	OTU numbers
NPK-1	69301	136	31072	1290
NPK-2	67852	158	28586	1322
NPK-3	32120	86	31063	1231
NPKM1-1	112068	238	27498	789
NPKM1-2	91771	211	24926	829
NPKM1-3	97281	216	30605	798
NPKM2-1	96341	195	34059	641
NPKM2-2	77822	229	27793	941
NPKM2-3	121493	245	32163	1167
NPKM3-1	128998	279	30423	1077
NPKM3-2	127252	302	35806	1049
NPKM3-3	104068	292	36150	1361

Table S2. Overall one-way ANOVA and assumption-check statistics for variables analyzed across fertilization treatments.

Variable	F (3, 8)	ANOVA p	Shapiro–Wilk p	Levene p
pH	39.0520	4.00e-05	0.636354	0.349226
TC	5.8019	0.020915	0.561945	0.233615
TN	1.5958	0.265149	0.570168	0.280507
NH_4^+-N	22.8294	0.000282	0.49699	0.692314
NO_3^--N	6.4615	0.015683	0.627609	0.673341
TP	22.6862	0.000288	0.947855	0.449973
AP	123.9650	4.80e-07	0.247192	0.200398
TK	1.3999	0.311903	0.556805	0.556824
AK	9.7252	0.004801	0.436215	0.245840
C/N	0.2071	0.888683	0.810205	0.063377
C/P	18.8238	0.000554	0.987674	0.146560
N/P	18.3808	0.000601	0.945864	0.101347
phoC gene abundance	7.1944	0.011646	0.305836	0.261387
phoC OTU richness	23.5129	0.000254	0.236573	0.829148
phoC Shannon index	1.5302	0.279804	0.443351	0.566574
ACP activity	22.2659	0.000308	0.933161	0.354008
phoD gene abundance	46.0760	2.16e-05	0.403595	0.098391
phoD OTU richness	13.0912	0.001876	0.82692	0.060699
phoD Shannon index	6.9027	0.013077	0.47504	0.209607
ALP activity	140.6655	2.93e-07	0.158901	0.303504

Shapiro–Wilk tests were applied to ANOVA residuals, and Levene's test centered on group means was used to assess homogeneity of variances. For the assumption checks, $p > 0.05$ indicates that no significant departure from the corresponding assumption was detected.

microorganisms

Table S3 Global topological properties of the *phoC* and *phoD* association networks.

Networks	Nodes	Edges	Positive	Negative	Density	Mean degree	Mean clustering coefficient
phoC	85	246	216 (87.80%)	30 (12.20%)	0.069	5.788	0.391
phoD	201	1093	600 (54.89%)	493 (45.11%)	0.054	10.876	0.437

Table S4 The correlation between soil properties, phosphatase activities and dominant *phoC*- and *phoD*-harboring bacterial genera. Only data with significant correlation relationships are retained.

	Genus	pH	TC	NH_4^+-N	NO_3^--N	TP	AP	TK	C/N	C/P	N/P	ACP
phoC	*Cupriavidus*	0.622*	0.769**	-0.643*	0.524	0.545	0.629*	-0.273	0.252	-0.531	-0.650*	0.657*
	Stenotrophomonas	-0.077	-0.329	0.154	-0.699*	-0.042	-0.021	0.056	0.077	0.056	0.014	0.091
	Serratia	0.382	0.165	-0.287	0.357	0.385	0.315	0.683*	-0.539	-0.420	-0.214	0.263
		pH	TC	NH_4^+-N	NO_3^--N	TP	AP	TK	C/N	C/P	N/P	ALP
phoD	*Bradyrhizobium*	-0.168	-0.420	0.301	-0.734**	-0.294	-0.203	-0.196	-0.140	0.287	0.308	-0.007
	Variibacter	-0.084	0.049	-0.077	0.685*	-0.161	-0.133	0.322	-0.035	0.126	0.182	0.021
	Pleomorphomonas	0.797**	0.224	-0.769**	-0.266	0.720**	0.818**	-0.063	0.140	-0.713**	-0.804**	0.643*
	Streptomyces	-0.077	-0.007	0.168	0.196	-0.021	-0.112	0.664*	-0.280	-0.014	0.210	-0.098
	Cupriavidus	-0.420	-0.573	0.538	-0.671*	-0.420	-0.462	-0.182	-0.161	0.469	0.434	-0.462
	Collimonas	-0.769**	-0.531	0.608*	0.063	-0.678*	-0.762**	-0.105	-0.077	0.685*	0.671*	-0.762**
	Amycolatopsis	-0.049	0.490	0.063	0.056	0.028	0.028	-0.434	0.608*	0.035	-0.168	0.140

Correlations were determined by Spearman's correlation. ** indicates significant correlations at $p < 0.01$; * indicates significantly correlations at $p < 0.05$. TC: soil total carbon; NH_4^+-N: ammonium nitrogen; NO_3^--N: Nitrate nitrogen; TP: total phosphorus; AP: available phosphorus; TK: total potassium; C/N ratio: soil total carbon /total nitrogen; C/P ratio: soil total carbon /total phosphorus; N/P ratio: total nitrogen/total phosphorus; ACP: acid phosphatase; ALP: alkaline phosphatase.

Table S5. The values of ACP and ALP activity, *phoC* and *phoD* gene copy number, OTU number and Shannon index of *phoC*- and *phoD*- harboring bacteria in soils with different long-term (10 years) manure addition treatments

	NPK	NPKM1	NPKM2	NPKM3
ACP activity (μg *p*NP g^{-1} soil h^{-1})	113.54±2.41 c	118.97±3.55 bc	124.9±3.51 b	142.61±7.42 a
ALP activity (μg *p*NP g^{-1} soil h^{-1})	45.17±1.86 b	55.12±6.65 b	55.41±5.90 b	127.48±6.42 a
phoC gene copies (10^7 g^{-1} soil)	1.19±0.10 b	1.51±0.14 b	1.53±0.22 b	1.92±0.27 a
phoD gene copies (10^7 g^{-1} soil)	5.02±0.67 d	8.72±0.75 c	11.85±1.19 b	20.08±2.88 a
OTU number (*phoC*)	91.67±17.39 c	157±16.46 b	162.33±19.35 b	207.33±14.5 a
Shannon index (*phoC*)	3.14±0.58 a	3.8±0.58 a	3.27±0.39 a	3.78±0.30 a
OTU number (*phoD*)	701±38.04 a	418±15.59 b	468.33±108.25 b	628.33±52.7 a
Shannon index (*phoD*)	6.9±0.07 a	6.16±0.51 b	5.77±0.47 b	7.02±0.37 a

Data are means ± standard deviation (n = 3 independent field plots). Different lowercase letters within row indicate significant differences among fertilization treatments at $p < 0.05$.

Table S6. The coordination of PCoA of *phoC*- and *phoD*-harboring bacteria

Gene	Treatment	PCoA1	PCoA2
phoC	NPK	-0.1671	-0.0561
phoC	NPK	-0.3447	0.1048
phoC	NPK	-0.3615	0.1379
phoC	NPKM1	-0.0059	0.2456
phoC	NPKM1	0.0171	-0.0861
phoC	NPKM1	0.007949	-0.3273
phoC	NPKM2	0.1053	-0.1677
phoC	NPKM2	0.146	-0.2767
phoC	NPKM2	0.2402	-0.0626
phoC	NPKM3	0.2018	0.003387
phoC	NPKM3	0.1396	0.4366
phoC	NPKM3	0.1953	0.2082
phoD	NPK	-0.41	0.1691
phoD	NPK	-0.4181	0.1471
phoD	NPK	-0.4134	0.1205
phoD	NPKM1	0.1445	-0.1349
phoD	NPKM1	0.1183	-0.1394
phoD	NPKM1	0.0175	-0.1245
phoD	NPKM2	-0.0515	-0.5697
phoD	NPKM2	0.1673	-0.0783
phoD	NPKM2	0.2044	0.1363
phoD	NPKM3	0.2219	0.1313
phoD	NPKM3	0.2422	0.1785
phoD	NPKM3	0.2769	0.1639

microorganisms

Table S7. The putative network hub of *phoC* - and *phoD* -harboring bacteria

Id	Network	No	Genus	Hub Score	Abundance	Degree
otu2178	*phoC*	1	*Cupriavidus*	0.9588235	0.0100	15
otu1072	*phoC*	2	*Cupriavidus*	0.9294118	0.0098	11
otu1047	*phoC*	3	*Cupriavidus*	0.9235294	0.0121	11
otu44904	*phoD*	1	*Collimonas*	0.9726368	0.0327	34
otu15406	*phoD*	2	*Pleomorphomonas*	0.9651741	0.0774	37
otu49281	*phoD*	3	*Bradyrhizobium*	0.960199	0.0615	31
otu5811	*phoD*	4	*Bradyrhizobium*	0.9552239	0.0179	32
otu14491	*phoD*	5	*Bradyrhizobium*	0.9477612	0.2341	28
otu62964	*phoD*	6	*Amycolatopsis*	0.9452736	0.2064	27

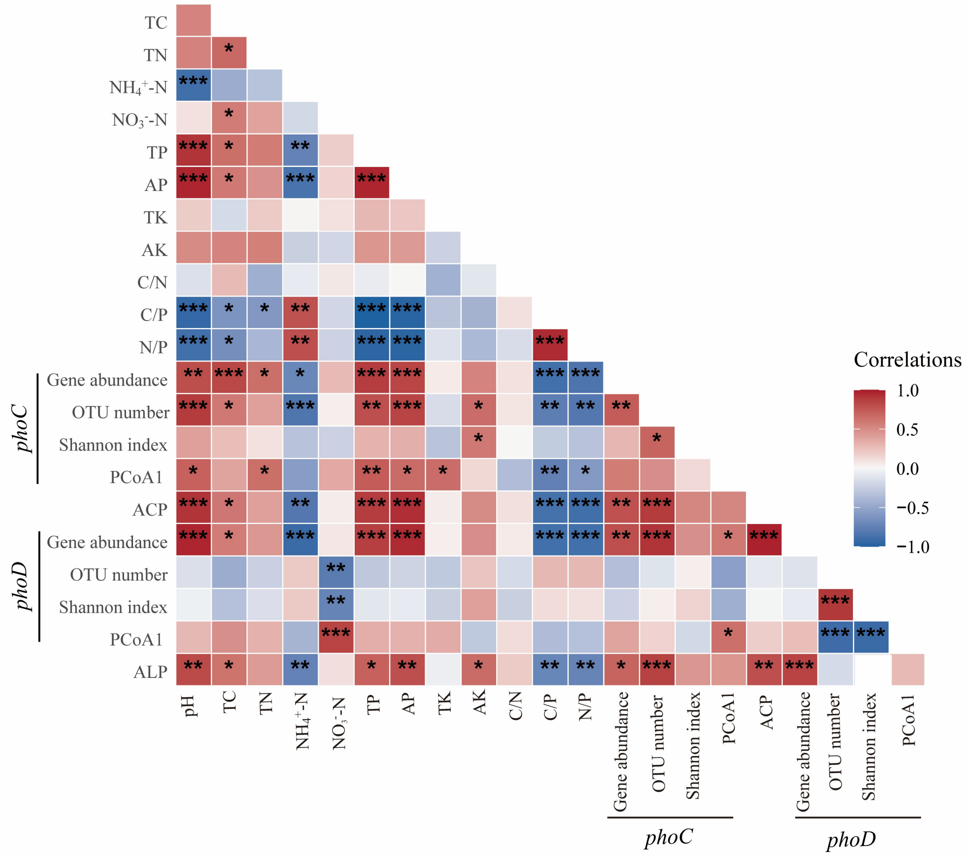

Figure S1 Correlation analysis between soil properties, phosphatase activities, gene abundance, α-diversity (OTU number and Shannon index), and bacterial community composition (PCoA1) were evaluated using Spearman's correlation. *$p < 0.05$; **$p < 0.01$; ***$p < 0.001$.

TC: soil total carbon; TN: total nitrogen; NH_4^+-N: ammonium nitrogen; NO_3^--N: nitrate nitrogen; TP: total phosphorus; AP: available phosphorus; TK: total potassium; AK: available potassium. C/N ratio: soil total carbon /total nitrogen; C/P ratio: soil total carbon /total phosphorus; N/P ratio: total nitrogen/total phosphorus.
